# (18-Crown-6)potassium [(1,2,5,6-η)-cyclo­octa-1,5-diene][(1,2,3,4-η)-naph­tha­lene]­ferrate(−I)

**DOI:** 10.1107/S160053681203704X

**Published:** 2012-09-01

**Authors:** William W. Brennessel, John E. Ellis

**Affiliations:** a Department of Chemistry, 207 Pleasant Street SE, University of Minnesota, Minneapolis, MN 55455, USA

## Abstract

The title salt, [K(C_12_H_24_O_6_)][Fe(C_8_H_12_)(C_10_H_8_)], is the only known naphthalene complex containing iron in a formally negative oxidation state. Each (naphthalene)(1,5-cod)ferrate(−I) anion is in contact with one (18-crown-6)potassium cation *via* K⋯C contacts to the outer four carbon atoms of the naphthalene ligand (cod = 1,5-cyclo­octa­diene, 18-crown-6 = 1,4,7,10,13,16-hexa­oxacyclo­octa­deca­ne). When using the midpoints of the coordinating olefin bonds, the overall geometry of the coordination sphere around iron can be best described as distorted tetra­hedral. The naphthalene fold angle between the plane of the iron-coordinating butadiene unit and the plane containing the *exo*-benzene moiety is 19.2 (1)°.

## Related literature

For the known complexes that contain iron in a formally negative oxidation state with solely olefinic ligands, see: Jonas (1979 ▶, 1981 ▶); Jonas *et al.* (1979 ▶); Jonas & Krüger (1980 ▶); Brennessel *et al.* (2007 ▶). For the various syntheses of the cobalt analog of the title complex, see: Brennessel *et al.* (2006 ▶); Brennessel & Ellis (2012 ▶). For an example of a diamagnetic, formally Fe(0) naphthalene­ferrate(−I), see: Schnökelborg *et al.* (2012 ▶). For details of the preparation and purification of reagents and solvents, and for descriptions of the equipment and techniques, see: Brennessel (2009 ▶). For a discussion of polyaromatic hydro­carbons and their Dewar’s resonance energies, see: Milun *et al.* (1972 ▶).
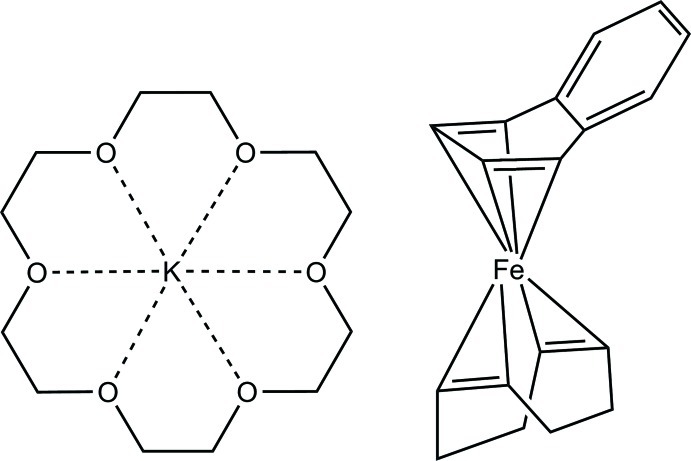


## Experimental

### 

#### Crystal data

[K(C_12_H_24_O_6_)][Fe(C_8_H_12_)(C_10_H_8_)]
*M* *_r_* = 595.60
Triclinic, 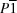

*a* = 9.244 (1) Å
*b* = 10.5285 (12) Å
*c* = 15.971 (2) Å
α = 76.085 (2)°
β = 89.651 (2)°
γ = 74.949 (2)°
*V* = 1454.4 (3) Å^3^
*Z* = 2
Mo *K*α radiation
μ = 0.70 mm^−1^
*T* = 173 K
0.42 × 0.32 × 0.22 mm

#### Data collection

Siemens SMART CCD diffractometer
Absorption correction: multi-scan (*SADABS*; Sheldrick, 2008*a* ▶) *T*_min_ = 0.666, *T*_max_ = 0.746
17413 measured reflections
6610 independent reflections
5576 reflections with *I* > 2σ(*I*)
*R*_int_ = 0.025

#### Refinement

*R*[*F* ^2^ > 2σ(*F* ^2^)] = 0.031
*wR*(*F* ^2^) = 0.073
*S* = 1.04
6610 reflections
423 parameters
H atoms treated by a mixture of independent and constrained refinement
Δρ_max_ = 0.32 e Å^−3^
Δρ_min_ = −0.29 e Å^−3^

### 

Data collection: *SMART* (Bruker, 2003 ▶); cell refinement: *SAINT* (Bruker, 2003 ▶); data reduction: *SAINT*; program(s) used to solve structure: *SIR97* (Altomare *et al.*, 1999 ▶); program(s) used to refine structure: *SHELXL97* (Sheldrick, 2008*b* ▶); molecular graphics: *SHELXTL* (Sheldrick, 2008*b* ▶); software used to prepare material for publication: *SHELXTL*.

## Supplementary Material

Crystal structure: contains datablock(s) I, global. DOI: 10.1107/S160053681203704X/wm2674sup1.cif

Structure factors: contains datablock(s) I. DOI: 10.1107/S160053681203704X/wm2674Isup2.hkl

Additional supplementary materials:  crystallographic information; 3D view; checkCIF report

## Figures and Tables

**Fig. 1.**
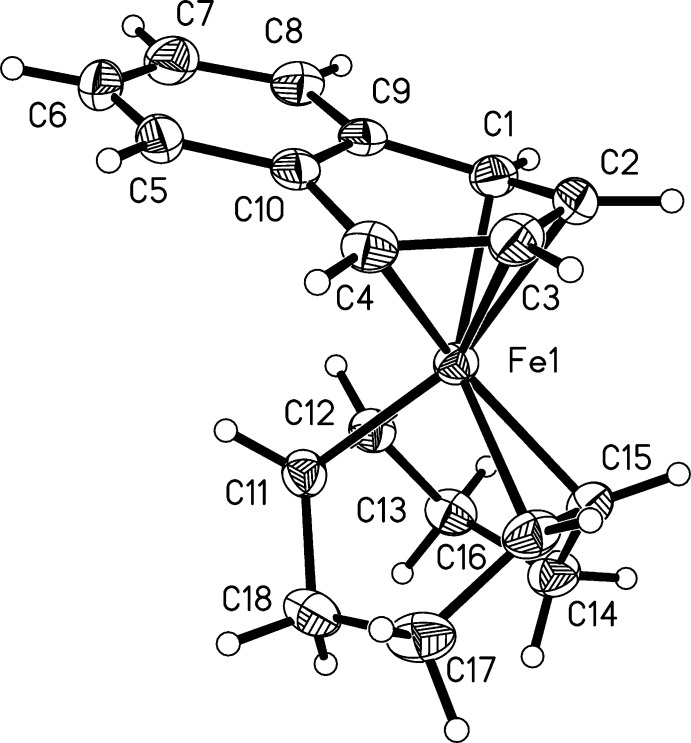
Molecular structure of the anion showing displacement ellipsoids at the 50% probability level.

**Fig. 2.**
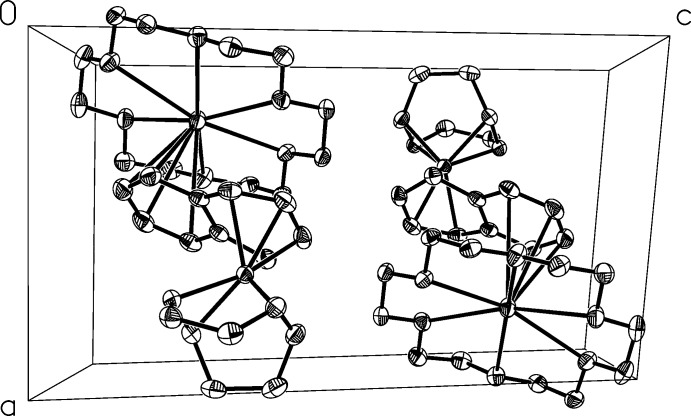
Unit cell packing plot that features the cation-anion contacts.

## supplementary crystallographic information

### Comment

To date there are very few reported complexes of iron in a formally negative oxidation state and supported solely by olefinic ligands. In the 1970s Klaus Jonas and coworkers devised a way to synthesize (*L*)_2_Li_2_Fe(C═C)_2_, where *L* = tetramethylethylenediamine and C═C = 2(ethylene) or 1,5-cyclooctadiene (cod) or *L* = 1,2-dimethoxyethane (dme) and C═C = 1,5-cod (Jonas, 1979, 1981; Jonas *et al.*, 1979; Jonas & Krüger, 1980), for which the cod complex is a direct derivative of the ethylene complex. Because ferrocene (FeCp_2_) was the starting material, the synthesis of the homoleptic ethylene complex required pressurized ethylene gas to fully displace both cyclopentadienyl ligands (5 atm prior to heating to 323 K in a closed vessel; Jonas, 1979). To avoid the need for the superambient pressures necessary with ferrocene, we devised syntheses from a ferrous halide, FeBr_2_. Recently we reported the syntheses of new ferrate anions bis(anthracene)ferrate(-I), bis(butadiene)ferrate(-I), and mixed-ligand (anthracene)(1,5-cod)ferrate(-I) (Brennessel *et al.*, 2007). The title complex is unique because it is the sole example of a naphthalene complex containing iron in a formally negative oxidation state. Only one other naphthaleneferrate(-I) has been reported in the primary literature, a diamagnetic formally Fe(0) complex, (18-crown-6)potassium (η^5^-C_5_Me_5_)(η^4^-naphthalene)ferrate(-I) (Schnökelborg *et al.*, 2012). Several neutral and cationic heteroleptic naphthalene—iron complexes have also been structurally characterized, as discussed elsewhere (Brennessel, 2009).

Unlike what was observed in the cobalt system, in which the reduction of CoBr_2_ by three equivalents of potassium naphthalene in the presence of excess 1,5-cod led to the homoleptic 1,5-cod anion [Co(η^4^-1,5-cod)_2_]^-^ (Brennessel *et al.*, 2006; Brennessel & Ellis, 2012), only one molecule of 1,5-cod is found coordinating to the iron atom regardless of excess 1,5-cod. This was not the only product, since carbonylation of the bulk material showed *ν*_CO_ stretching frequencies corresponding to [Fe_2_(CO)_8_]^2-^ (major) and [Fe(CO)_4_]^2-^ (minor). If the naphthalene radical anion is reducing enough to afford an Fe(-II) species directly, then that species could be the precursor to the minor carbonylation product, the Fe(-II) carbonyl. However, since the yield of the title complex is modest (40–50%), there is likely excess reducing agent left over from the initial reduction which easily could have reduced the Fe(-I) carbonyl to Fe(-II). Unfortunately, it has proved very difficult to separate the title complex from the naphthalene radical anion, and no further optimizations or characterizations have been performed to date.

The bond lengths of the metal-coordinating olefins (C1═C2 and C3═C4, Figure 1) of the naphthalene ligand (1.424 (3) Å, avg.) are statistically identical to those found in the related anthracene-cod ferrate anion, [Fe(C_14_H_10_)(C_8_H_12_)]^-^ (1.422 (4) Å, avg.; Brennessel *et al.*, 2007), which suggests that naphthalene is performing an equivalent role in supporting the low-valent iron atom. Even so, anthracene quantitatively displaces naphthalene at room temperature in THF solution (*i.e.*, the title complex can be converted to the anthracene-cod ferrate with ease), a result that can be justified with Dewar's resonance energies (Milun *et al.*, 1972). Both the title complex and the anthracene-cod ferrate have an essentially tetrahedral geometry about their iron atoms and have similar polyaromatic hydrocarbon fold angles (for the title structure the fold angle between the planes defined by atoms C1, C2, C3, C4 and C1, C4, C5, C6, C7, C8, C9, C10, respectively, amounts to 19.2 (1) °.)

The packing of the molecular entities is shown in Figure 2.

### Experimental

Details on the preparation and purification of reagents and solvents, and descriptions of the equipment and techniques can be found elsewhere (Brennessel, 2009). Under argon, an orange slurry of anhydrous FeBr_2_ (0.500 g, 2.32 mmol) in THF (50 ml, 195 K) was added to a deep green solution of K[C_10_H_8_] (6.86 mmol) and excess 1,5-cyclooctadiene (0.882 g, 8.15 mmol) in THF (50 ml, 195 K). The resulting reddish-yellow solution was warmed slowly to room temperature, when it was filtered to remove KBr. 18-crown-6 (0.613 g, 2.32 mmol) in THF (30 ml) was added to the deep red filtrate and the solvent was removed *in vacuo*. Pentane (40 ml) was added and the solid was carefully scraped off the flask wall with the stir bar. The slurry was then filtered, and the product was washed with pentane (30 ml) and dried *in vacuo*, yielding a dark red solid (0.607 g, 44% assuming the uni-negative title complex: see Comment above). An analytically pure bulk sample has not been obtained to date. Dark red blocks were grown from a pentane-layered THF solution at 273 K.

### Refinement

Hydrogen atoms on the naphthalene ligand and on the metal-coordinating carbon atoms of the 1,5-cod ligand were found from a difference Fourier map, and their positional and isotropic displacement parameters were refined independently from those of their respective bonded carbon atoms. All other hydrogen atoms were placed geometrically, and refined relative to their respective bonded carbon atoms with a bond lengths of 0.99 Å and *U*_iso_[H] = 1.2^.^*U*_eq_[C].

### Crystal data

**Table:**

[K(C_12_H_24_O_6_)][Fe(C_8_H_12_)(C_10_H_8_)]	*Z* = 2
*M**_r_* = 595.60	*F*(000) = 634
Triclinic, *P*1	*D*_x_ = 1.360 Mg m^−^^3^
Hall symbol: -P 1	Mo *K*α radiation, λ = 0.71073 Å
*a* = 9.244 (1) Å	Cell parameters from 3650 reflections
*b* = 10.5285 (12) Å	θ = 2.3–27.5°
*c* = 15.971 (2) Å	µ = 0.70 mm^−^^1^
α = 76.085 (2)°	*T* = 173 K
β = 89.651 (2)°	Block, dark red
γ = 74.949 (2)°	0.42 × 0.32 × 0.22 mm
*V* = 1454.4 (3) Å^3^	

### Data collection

**Table:**

Siemens SMART CCD diffractometer	6610 independent reflections
Radiation source: normal-focus sealed tube	5576 reflections with *I* > 2σ(*I*)
Graphite monochromator	*R*_int_ = 0.025
ω scans per φ	θ_max_ = 27.5°, θ_min_ = 1.3°
Absorption correction: multi-scan (*SADABS*; Sheldrick, 2008*a*)	*h* = −12→11
*T*_min_ = 0.666, *T*_max_ = 0.746	*k* = −13→13
17413 measured reflections	*l* = −20→20

### Refinement

**Table:**

Refinement on *F*^2^	Primary atom site location: structure-invariant direct methods
Least-squares matrix: full	Secondary atom site location: difference Fourier map
*R*[*F*^2^ > 2σ(*F*^2^)] = 0.031	Hydrogen site location: inferred from neighbouring sites
*wR*(*F*^2^) = 0.073	H atoms treated by a mixture of independent and constrained refinement
*S* = 1.04	*w* = 1/[σ^2^(*F*_o_^2^) + (0.0321*P*)^2^ + 0.4038*P*] where *P* = (*F*_o_^2^ + 2*F*_c_^2^)/3
6610 reflections	(Δ/σ)_max_ = 0.001
423 parameters	Δρ_max_ = 0.32 e Å^−^^3^
0 restraints	Δρ_min_ = −0.29 e Å^−^^3^

### Special details

**Table:**

Geometry. All e.s.d.'s (except the e.s.d. in the dihedral angle between two l.s. planes) are estimated using the full covariance matrix. The cell e.s.d.'s are taken into account individually in the estimation of e.s.d.'s in distances, angles and torsion angles; correlations between e.s.d.'s in cell parameters are only used when they are defined by crystal symmetry. An approximate (isotropic) treatment of cell e.s.d.'s is used for estimating e.s.d.'s involving l.s. planes.
Refinement. Refinement of *F*^2^ against ALL reflections. The weighted *R*-factor *wR* and goodness of fit *S* are based on *F*^2^, conventional *R*-factors *R* are based on *F*, with *F* set to zero for negative *F*^2^. The threshold expression of *F*^2^ > σ(*F*^2^) is used only for calculating *R*-factors(gt) *etc*. and is not relevant to the choice of reflections for refinement. *R*-factors based on *F*^2^ are statistically about twice as large as those based on *F*, and *R*- factors based on ALL data will be even larger.

### Fractional atomic coordinates and isotropic or equivalent isotropic displacement parameters (Å^2^)

**Table:**

	*x*	*y*	*z*	*U*_iso_*/*U*_eq_	
Fe1	0.66405 (2)	0.07626 (2)	0.327243 (13)	0.02107 (7)	
C1	0.42748 (18)	0.15429 (16)	0.29524 (11)	0.0273 (3)	
H1	0.364 (2)	0.1109 (18)	0.2718 (11)	0.033 (5)*	
C2	0.46028 (19)	0.12384 (16)	0.38572 (11)	0.0300 (4)	
H2	0.420 (2)	0.058 (2)	0.4266 (13)	0.041 (5)*	
C3	0.5629 (2)	0.18225 (16)	0.41542 (11)	0.0296 (4)	
H3	0.597 (2)	0.1600 (19)	0.4752 (13)	0.036 (5)*	
C4	0.62680 (19)	0.27169 (16)	0.35391 (10)	0.0271 (3)	
H4	0.704 (2)	0.3085 (17)	0.3709 (11)	0.029 (5)*	
C5	0.57720 (19)	0.45705 (16)	0.21584 (11)	0.0292 (4)	
H5	0.644 (2)	0.4972 (18)	0.2374 (11)	0.029 (5)*	
C6	0.5125 (2)	0.50782 (18)	0.13140 (12)	0.0347 (4)	
H6	0.535 (2)	0.583 (2)	0.0952 (12)	0.038 (5)*	
C7	0.4159 (2)	0.44642 (19)	0.10107 (12)	0.0363 (4)	
H7	0.370 (2)	0.4835 (19)	0.0440 (13)	0.038 (5)*	
C8	0.38090 (19)	0.33404 (17)	0.15534 (11)	0.0304 (4)	
H8	0.312 (2)	0.2921 (18)	0.1354 (11)	0.033 (5)*	
C9	0.44646 (17)	0.27912 (15)	0.23910 (10)	0.0245 (3)	
C10	0.54882 (17)	0.34143 (15)	0.27027 (10)	0.0241 (3)	
C11	0.83151 (18)	0.08378 (17)	0.24371 (10)	0.0252 (3)	
H11	0.8227 (19)	0.1757 (18)	0.2129 (11)	0.029 (5)*	
C12	0.72907 (18)	0.02003 (16)	0.21617 (10)	0.0249 (3)	
H12	0.658 (2)	0.0732 (18)	0.1659 (11)	0.030 (5)*	
C13	0.7681 (2)	−0.13215 (17)	0.22562 (11)	0.0291 (4)	
H13A	0.681 (2)	−0.1568 (18)	0.2049 (11)	0.033 (5)*	
H13B	0.853 (2)	−0.1600 (18)	0.1890 (12)	0.034 (5)*	
C14	0.8100 (2)	−0.21363 (17)	0.32012 (12)	0.0311 (4)	
H14A	0.916 (2)	−0.2459 (18)	0.3285 (11)	0.032 (5)*	
H14B	0.772 (2)	−0.2956 (19)	0.3322 (12)	0.036 (5)*	
C15	0.74815 (18)	−0.12962 (16)	0.38461 (10)	0.0274 (3)	
H15	0.6795 (19)	−0.1666 (17)	0.4243 (11)	0.025 (4)*	
C16	0.83112 (19)	−0.05207 (17)	0.41517 (11)	0.0294 (4)	
H16	0.813 (2)	−0.0428 (18)	0.4740 (12)	0.035 (5)*	
C17	0.9833 (2)	−0.0384 (2)	0.38516 (12)	0.0369 (4)	
H17A	1.063 (2)	−0.122 (2)	0.4099 (12)	0.042 (5)*	
H17B	1.006 (2)	0.033 (2)	0.4088 (12)	0.037 (5)*	
C18	0.98569 (19)	0.00363 (19)	0.28624 (12)	0.0321 (4)	
H18A	1.0232 (19)	−0.0742 (18)	0.2615 (11)	0.030 (5)*	
H18B	1.058 (2)	0.0561 (19)	0.2702 (12)	0.038 (5)*	
K1	0.20189 (4)	0.61844 (4)	0.21260 (2)	0.02749 (9)	
O1	0.15085 (12)	0.44660 (11)	0.36324 (7)	0.0284 (2)	
O2	−0.03471 (13)	0.48729 (11)	0.21687 (7)	0.0286 (2)	
O3	0.02462 (13)	0.65530 (12)	0.05729 (7)	0.0315 (3)	
O4	0.18316 (13)	0.85078 (12)	0.06839 (7)	0.0330 (3)	
O5	0.37379 (13)	0.80421 (11)	0.21515 (7)	0.0310 (3)	
O6	0.29972 (12)	0.64416 (11)	0.37056 (7)	0.0256 (2)	
C19	0.02914 (19)	0.38609 (17)	0.36605 (11)	0.0316 (4)	
H19A	−0.0635	0.4477	0.3797	0.038*	
H19B	0.0515	0.2998	0.4114	0.038*	
C20	0.00795 (19)	0.35982 (16)	0.27952 (11)	0.0308 (4)	
H20A	0.1025	0.3025	0.2644	0.037*	
H20B	−0.0710	0.3115	0.2808	0.037*	
C21	−0.06693 (19)	0.47181 (18)	0.13330 (11)	0.0310 (4)	
H21A	−0.1514	0.4298	0.1351	0.037*	
H21B	0.0219	0.4120	0.1145	0.037*	
C22	−0.10719 (19)	0.60868 (19)	0.07117 (11)	0.0332 (4)	
H22A	−0.1490	0.6027	0.0158	0.040*	
H22B	−0.1841	0.6730	0.0950	0.040*	
C23	−0.0027 (2)	0.78647 (17)	−0.00035 (11)	0.0355 (4)	
H23A	−0.0771	0.8532	0.0232	0.043*	
H23B	−0.0440	0.7849	−0.0570	0.043*	
C24	0.1409 (2)	0.82724 (18)	−0.01150 (11)	0.0358 (4)	
H24A	0.2202	0.7543	−0.0266	0.043*	
H24B	0.1270	0.9108	−0.0586	0.043*	
C25	0.31741 (19)	0.89400 (18)	0.06443 (11)	0.0342 (4)	
H25A	0.3073	0.9753	0.0160	0.041*	
H25B	0.4031	0.8211	0.0547	0.041*	
C26	0.3439 (2)	0.92658 (17)	0.14826 (11)	0.0330 (4)	
H26A	0.4303	0.9665	0.1451	0.040*	
H26B	0.2542	0.9933	0.1605	0.040*	
C30	0.17578 (18)	0.48077 (17)	0.44181 (10)	0.0278 (3)	
H30A	0.1836	0.4013	0.4912	0.033*	
H30B	0.0907	0.5555	0.4499	0.033*	
C31	0.31821 (18)	0.52392 (16)	0.43816 (10)	0.0272 (3)	
H31A	0.3414	0.5416	0.4940	0.033*	
H31B	0.4024	0.4511	0.4270	0.033*	
C32	0.43081 (17)	0.69279 (16)	0.36395 (10)	0.0267 (3)	
H32A	0.5150	0.6267	0.3462	0.032*	
H32B	0.4602	0.7039	0.4207	0.032*	
C33	0.39767 (18)	0.82636 (16)	0.29834 (11)	0.0283 (3)	
H33A	0.3070	0.8895	0.3128	0.034*	
H33B	0.4829	0.8672	0.2981	0.034*	

### Atomic displacement parameters (Å^2^)

**Table:**

	*U*^11^	*U*^22^	*U*^33^	*U*^12^	*U*^13^	*U*^23^
Fe1	0.02165 (12)	0.02075 (11)	0.01995 (11)	−0.00350 (8)	0.00137 (8)	−0.00573 (8)
C1	0.0217 (8)	0.0211 (8)	0.0376 (9)	−0.0045 (6)	0.0020 (7)	−0.0057 (7)
C2	0.0292 (9)	0.0237 (8)	0.0328 (9)	−0.0032 (7)	0.0117 (7)	−0.0034 (7)
C3	0.0367 (9)	0.0258 (8)	0.0231 (8)	−0.0009 (7)	0.0062 (7)	−0.0082 (7)
C4	0.0287 (8)	0.0261 (8)	0.0290 (8)	−0.0057 (7)	0.0028 (7)	−0.0130 (7)
C5	0.0276 (8)	0.0233 (8)	0.0380 (9)	−0.0077 (7)	0.0099 (7)	−0.0090 (7)
C6	0.0368 (10)	0.0258 (9)	0.0352 (10)	−0.0050 (7)	0.0134 (8)	0.0002 (7)
C7	0.0351 (10)	0.0371 (10)	0.0271 (9)	0.0003 (8)	0.0024 (8)	−0.0006 (8)
C8	0.0251 (8)	0.0314 (9)	0.0320 (9)	−0.0026 (7)	−0.0015 (7)	−0.0076 (7)
C9	0.0201 (7)	0.0213 (7)	0.0301 (8)	−0.0004 (6)	0.0048 (6)	−0.0085 (6)
C10	0.0229 (8)	0.0206 (7)	0.0289 (8)	−0.0021 (6)	0.0076 (6)	−0.0105 (6)
C11	0.0265 (8)	0.0252 (8)	0.0247 (8)	−0.0075 (6)	0.0057 (6)	−0.0072 (6)
C12	0.0248 (8)	0.0284 (8)	0.0213 (7)	−0.0048 (6)	0.0025 (6)	−0.0082 (6)
C13	0.0287 (9)	0.0295 (9)	0.0327 (9)	−0.0068 (7)	0.0040 (7)	−0.0157 (7)
C14	0.0304 (9)	0.0230 (8)	0.0389 (10)	−0.0042 (7)	0.0006 (7)	−0.0088 (7)
C15	0.0276 (8)	0.0239 (8)	0.0257 (8)	−0.0029 (7)	0.0000 (7)	−0.0009 (6)
C16	0.0296 (9)	0.0297 (9)	0.0244 (8)	−0.0009 (7)	−0.0048 (7)	−0.0054 (7)
C17	0.0275 (9)	0.0385 (10)	0.0422 (11)	−0.0033 (8)	−0.0098 (8)	−0.0112 (8)
C18	0.0213 (8)	0.0342 (9)	0.0443 (10)	−0.0077 (7)	0.0053 (7)	−0.0160 (8)
K1	0.03164 (19)	0.03177 (19)	0.02181 (17)	−0.01498 (15)	0.00019 (14)	−0.00468 (14)
O1	0.0298 (6)	0.0331 (6)	0.0255 (6)	−0.0146 (5)	0.0039 (5)	−0.0066 (5)
O2	0.0335 (6)	0.0279 (6)	0.0285 (6)	−0.0118 (5)	0.0009 (5)	−0.0107 (5)
O3	0.0300 (6)	0.0322 (6)	0.0298 (6)	−0.0080 (5)	−0.0047 (5)	−0.0032 (5)
O4	0.0394 (7)	0.0395 (7)	0.0219 (6)	−0.0168 (5)	0.0009 (5)	−0.0040 (5)
O5	0.0396 (7)	0.0238 (6)	0.0294 (6)	−0.0127 (5)	−0.0057 (5)	−0.0015 (5)
O6	0.0245 (6)	0.0247 (6)	0.0260 (6)	−0.0074 (4)	−0.0034 (4)	−0.0025 (4)
C19	0.0328 (9)	0.0314 (9)	0.0324 (9)	−0.0158 (7)	0.0047 (7)	−0.0033 (7)
C20	0.0303 (9)	0.0268 (8)	0.0388 (9)	−0.0132 (7)	0.0045 (7)	−0.0089 (7)
C21	0.0294 (9)	0.0406 (10)	0.0323 (9)	−0.0158 (7)	0.0051 (7)	−0.0196 (8)
C22	0.0258 (8)	0.0476 (11)	0.0297 (9)	−0.0094 (8)	−0.0020 (7)	−0.0164 (8)
C23	0.0460 (11)	0.0296 (9)	0.0270 (9)	−0.0037 (8)	−0.0110 (8)	−0.0063 (7)
C24	0.0544 (12)	0.0294 (9)	0.0215 (8)	−0.0106 (8)	−0.0001 (8)	−0.0028 (7)
C25	0.0330 (9)	0.0304 (9)	0.0340 (9)	−0.0094 (7)	0.0034 (7)	0.0031 (7)
C26	0.0327 (9)	0.0252 (8)	0.0382 (10)	−0.0126 (7)	−0.0038 (7)	0.0033 (7)
C30	0.0311 (9)	0.0301 (8)	0.0198 (8)	−0.0080 (7)	0.0016 (6)	−0.0018 (6)
C31	0.0292 (8)	0.0284 (8)	0.0209 (8)	−0.0050 (7)	−0.0024 (6)	−0.0028 (6)
C32	0.0229 (8)	0.0299 (8)	0.0288 (8)	−0.0075 (6)	−0.0032 (6)	−0.0096 (7)
C33	0.0256 (8)	0.0268 (8)	0.0352 (9)	−0.0096 (7)	−0.0019 (7)	−0.0095 (7)

### Geometric parameters (Å, º)

**Table:**

Fe1—C12	2.0410 (15)	C18—H18B	0.972 (19)
Fe1—C11	2.0421 (15)	K1—O1	2.7604 (11)
Fe1—C16	2.0525 (16)	K1—O6	2.7818 (11)
Fe1—C3	2.0736 (16)	K1—O5	2.8294 (11)
Fe1—C15	2.0786 (16)	K1—O2	2.8664 (11)
Fe1—C2	2.0940 (16)	K1—O3	2.8715 (12)
Fe1—C4	2.1413 (16)	K1—O4	2.8972 (12)
Fe1—C1	2.1441 (16)	O1—C30	1.4216 (19)
C1—C2	1.420 (2)	O1—C19	1.4254 (19)
C1—C9	1.454 (2)	O2—C21	1.4252 (19)
C1—H1	0.960 (18)	O2—C20	1.4289 (19)
C2—C3	1.397 (2)	O3—C23	1.424 (2)
C2—H2	0.98 (2)	O3—C22	1.425 (2)
C3—C4	1.427 (2)	O4—C25	1.425 (2)
C3—H3	0.961 (19)	O4—C24	1.432 (2)
C4—C10	1.452 (2)	O5—C26	1.4263 (18)
C4—H4	0.961 (18)	O5—C33	1.4310 (19)
C5—C6	1.400 (3)	O6—C31	1.4247 (18)
C5—C10	1.400 (2)	O6—C32	1.4271 (18)
C5—K1	3.4387 (17)	C19—C20	1.497 (2)
C5—H5	0.941 (18)	C19—H19A	0.9900
C6—C7	1.380 (3)	C19—H19B	0.9900
C6—K1	3.1978 (18)	C20—H20A	0.9900
C6—H6	0.932 (19)	C20—H20B	0.9900
C7—C8	1.399 (2)	C21—C22	1.496 (2)
C7—K1	3.1647 (19)	C21—H21A	0.9900
C7—H7	0.955 (19)	C21—H21B	0.9900
C8—C9	1.395 (2)	C22—H22A	0.9900
C8—K1	3.3565 (18)	C22—H22B	0.9900
C8—H8	0.959 (18)	C23—C24	1.494 (3)
C9—C10	1.435 (2)	C23—H23A	0.9900
C11—C12	1.420 (2)	C23—H23B	0.9900
C11—C18	1.522 (2)	C24—H24A	0.9900
C11—H11	0.957 (18)	C24—H24B	0.9900
C12—C13	1.518 (2)	C25—C26	1.496 (2)
C12—H12	0.994 (18)	C25—H25A	0.9900
C13—C14	1.541 (2)	C25—H25B	0.9900
C13—H13A	0.986 (18)	C26—H26A	0.9900
C13—H13B	0.997 (19)	C26—H26B	0.9900
C14—C15	1.529 (2)	C30—C31	1.497 (2)
C14—H14A	0.944 (19)	C30—H30A	0.9900
C14—H14B	0.993 (19)	C30—H30B	0.9900
C15—C16	1.419 (2)	C31—H31A	0.9900
C15—H15	0.980 (17)	C31—H31B	0.9900
C16—C17	1.514 (3)	C32—C33	1.497 (2)
C16—H16	0.977 (19)	C32—H32A	0.9900
C17—C18	1.537 (3)	C32—H32B	0.9900
C17—H17A	0.98 (2)	C33—H33A	0.9900
C17—H17B	0.99 (2)	C33—H33B	0.9900
C18—H18A	0.977 (18)		
			
C12—Fe1—C11	40.72 (6)	C18—C17—H17B	108.4 (11)
C12—Fe1—C16	101.66 (7)	H17A—C17—H17B	106.2 (16)
C11—Fe1—C16	85.08 (7)	C11—C18—C17	112.01 (14)
C12—Fe1—C3	163.78 (7)	C11—C18—H18A	108.9 (10)
C11—Fe1—C3	137.43 (7)	C17—C18—H18A	112.3 (10)
C16—Fe1—C3	93.69 (7)	C11—C18—H18B	110.4 (11)
C12—Fe1—C15	83.82 (7)	C17—C18—H18B	109.6 (11)
C11—Fe1—C15	94.47 (7)	H18A—C18—H18B	103.1 (15)
C16—Fe1—C15	40.18 (6)	O1—K1—O6	60.69 (3)
C3—Fe1—C15	111.66 (7)	O1—K1—O5	121.15 (3)
C12—Fe1—C2	135.35 (7)	O6—K1—O5	60.68 (3)
C11—Fe1—C2	162.51 (7)	O1—K1—O2	59.38 (3)
C16—Fe1—C2	111.20 (7)	O6—K1—O2	116.23 (3)
C3—Fe1—C2	39.17 (7)	O5—K1—O2	165.04 (4)
C15—Fe1—C2	102.14 (7)	O1—K1—O3	119.37 (3)
C12—Fe1—C4	128.82 (6)	O6—K1—O3	162.63 (4)
C11—Fe1—C4	100.06 (6)	O5—K1—O3	117.93 (3)
C16—Fe1—C4	105.99 (7)	O2—K1—O3	60.05 (3)
C3—Fe1—C4	39.54 (6)	O1—K1—O4	163.85 (4)
C15—Fe1—C4	141.78 (6)	O6—K1—O4	115.14 (3)
C2—Fe1—C4	70.01 (6)	O5—K1—O4	58.73 (3)
C12—Fe1—C1	99.01 (7)	O2—K1—O4	116.06 (3)
C11—Fe1—C1	126.64 (6)	O3—K1—O4	59.37 (3)
C16—Fe1—C1	146.94 (7)	C30—O1—C19	112.41 (12)
C3—Fe1—C1	69.83 (7)	C30—O1—K1	116.80 (9)
C15—Fe1—C1	118.39 (6)	C19—O1—K1	120.44 (9)
C2—Fe1—C1	39.12 (7)	C21—O2—C20	111.93 (12)
C4—Fe1—C1	79.83 (6)	C21—O2—K1	110.68 (9)
C2—C1—C9	120.56 (15)	C20—O2—K1	108.68 (9)
C2—C1—Fe1	68.54 (9)	C23—O3—C22	112.93 (13)
C9—C1—Fe1	92.17 (10)	C23—O3—K1	114.91 (9)
C2—C1—H1	120.7 (11)	C22—O3—K1	114.12 (9)
C9—C1—H1	115.8 (11)	C25—O4—C24	112.73 (13)
Fe1—C1—H1	126.7 (11)	C25—O4—K1	111.39 (9)
C3—C2—C1	118.02 (15)	C24—O4—K1	112.03 (9)
C3—C2—Fe1	69.62 (9)	C26—O5—C33	112.31 (12)
C1—C2—Fe1	72.35 (9)	C26—O5—K1	118.54 (9)
C3—C2—H2	120.0 (11)	C33—O5—K1	114.97 (9)
C1—C2—H2	121.8 (11)	C31—O6—C32	111.88 (11)
Fe1—C2—H2	124.6 (11)	C31—O6—K1	113.19 (8)
C2—C3—C4	118.71 (15)	C32—O6—K1	112.13 (9)
C2—C3—Fe1	71.20 (9)	O1—C19—C20	108.19 (13)
C4—C3—Fe1	72.78 (9)	O1—C19—H19A	110.1
C2—C3—H3	122.6 (11)	C20—C19—H19A	110.1
C4—C3—H3	118.6 (11)	O1—C19—H19B	110.1
Fe1—C3—H3	123.8 (11)	C20—C19—H19B	110.1
C3—C4—C10	120.06 (15)	H19A—C19—H19B	108.4
C3—C4—Fe1	67.67 (9)	O2—C20—C19	108.37 (13)
C10—C4—Fe1	92.85 (10)	O2—C20—H20A	110.0
C3—C4—H4	121.5 (10)	C19—C20—H20A	110.0
C10—C4—H4	116.0 (10)	O2—C20—H20B	110.0
Fe1—C4—H4	125.3 (10)	C19—C20—H20B	110.0
C6—C5—C10	120.95 (16)	H20A—C20—H20B	108.4
C6—C5—H5	121.4 (11)	O2—C21—C22	108.77 (13)
C10—C5—H5	117.6 (11)	O2—C21—H21A	109.9
K1—C5—H5	116.1 (11)	C22—C21—H21A	109.9
C7—C6—C5	120.30 (16)	O2—C21—H21B	109.9
C7—C6—H6	119.6 (12)	C22—C21—H21B	109.9
C5—C6—H6	120.1 (12)	H21A—C21—H21B	108.3
K1—C6—H6	106.6 (12)	O3—C22—C21	108.65 (13)
C6—C7—C8	119.87 (17)	O3—C22—H22A	110.0
C6—C7—H7	119.7 (12)	C21—C22—H22A	110.0
C8—C7—H7	120.4 (12)	O3—C22—H22B	110.0
K1—C7—H7	103.4 (12)	C21—C22—H22B	110.0
C9—C8—C7	121.13 (16)	H22A—C22—H22B	108.3
C9—C8—H8	118.3 (11)	O3—C23—C24	109.44 (14)
C7—C8—H8	120.6 (11)	O3—C23—H23A	109.8
K1—C8—H8	111.7 (11)	C24—C23—H23A	109.8
C8—C9—C10	119.06 (15)	O3—C23—H23B	109.8
C8—C9—C1	123.75 (15)	C24—C23—H23B	109.8
C10—C9—C1	116.98 (14)	H23A—C23—H23B	108.2
C5—C10—C9	118.62 (15)	O4—C24—C23	107.90 (14)
C5—C10—C4	124.29 (15)	O4—C24—H24A	110.1
C9—C10—C4	116.83 (14)	C23—C24—H24A	110.1
C12—C11—C18	122.17 (15)	O4—C24—H24B	110.1
C12—C11—Fe1	69.60 (9)	C23—C24—H24B	110.1
C18—C11—Fe1	112.75 (11)	H24A—C24—H24B	108.4
C12—C11—H11	116.2 (10)	O4—C25—C26	108.20 (14)
C18—C11—H11	116.0 (10)	O4—C25—H25A	110.1
Fe1—C11—H11	110.0 (10)	C26—C25—H25A	110.1
C11—C12—C13	121.97 (14)	O4—C25—H25B	110.1
C11—C12—Fe1	69.68 (9)	C26—C25—H25B	110.1
C13—C12—Fe1	113.82 (11)	H25A—C25—H25B	108.4
C11—C12—H12	117.4 (10)	O5—C26—C25	108.47 (13)
C13—C12—H12	113.5 (10)	O5—C26—H26A	110.0
Fe1—C12—H12	112.3 (10)	C25—C26—H26A	110.0
C12—C13—C14	112.40 (13)	O5—C26—H26B	110.0
C12—C13—H13A	109.6 (10)	C25—C26—H26B	110.0
C14—C13—H13A	108.0 (10)	H26A—C26—H26B	108.4
C12—C13—H13B	110.3 (10)	O1—C30—C31	108.80 (13)
C14—C13—H13B	109.5 (10)	O1—C30—H30A	109.9
H13A—C13—H13B	106.9 (14)	C31—C30—H30A	109.9
C15—C14—C13	112.34 (13)	O1—C30—H30B	109.9
C15—C14—H14A	110.1 (11)	C31—C30—H30B	109.9
C13—C14—H14A	109.2 (11)	H30A—C30—H30B	108.3
C15—C14—H14B	108.8 (11)	O6—C31—C30	108.64 (12)
C13—C14—H14B	110.3 (11)	O6—C31—H31A	110.0
H14A—C14—H14B	105.9 (15)	C30—C31—H31A	110.0
C16—C15—C14	122.00 (15)	O6—C31—H31B	110.0
C16—C15—Fe1	68.92 (9)	C30—C31—H31B	110.0
C14—C15—Fe1	113.32 (11)	H31A—C31—H31B	108.3
C16—C15—H15	116.8 (10)	O6—C32—C33	108.81 (12)
C14—C15—H15	114.3 (10)	O6—C32—H32A	109.9
Fe1—C15—H15	112.9 (10)	C33—C32—H32A	109.9
C15—C16—C17	124.39 (16)	O6—C32—H32B	109.9
C15—C16—Fe1	70.90 (9)	C33—C32—H32B	109.9
C17—C16—Fe1	110.35 (12)	H32A—C32—H32B	108.3
C15—C16—H16	115.1 (11)	O5—C33—C32	108.60 (12)
C17—C16—H16	114.7 (11)	O5—C33—H33A	110.0
Fe1—C16—H16	112.2 (11)	C32—C33—H33A	110.0
C16—C17—C18	112.64 (14)	O5—C33—H33B	110.0
C16—C17—H17A	111.2 (11)	C32—C33—H33B	110.0
C18—C17—H17A	111.3 (12)	H33A—C33—H33B	108.4
C16—C17—H17B	106.7 (11)		
			
C12—Fe1—C1—C2	161.21 (10)	C7—C6—K1—O3	−41.08 (10)
C11—Fe1—C1—C2	−165.77 (10)	C5—C6—K1—O3	−163.09 (10)
C16—Fe1—C1—C2	33.00 (16)	C7—C6—K1—O4	−99.33 (11)
C3—Fe1—C1—C2	−30.98 (10)	C5—C6—K1—O4	138.66 (11)
C15—Fe1—C1—C2	73.40 (11)	C5—C6—K1—C7	−122.01 (16)
C4—Fe1—C1—C2	−70.74 (10)	C7—C6—K1—C8	31.25 (10)
C12—Fe1—C1—C9	−76.67 (10)	C5—C6—K1—C8	−90.76 (11)
C11—Fe1—C1—C9	−43.65 (13)	C7—C6—K1—C5	122.01 (16)
C16—Fe1—C1—C9	155.12 (11)	C9—C8—K1—O1	−41.96 (10)
C3—Fe1—C1—C9	91.14 (11)	C7—C8—K1—O1	−165.52 (11)
C15—Fe1—C1—C9	−164.48 (9)	C9—C8—K1—O6	12.38 (11)
C2—Fe1—C1—C9	122.12 (14)	C7—C8—K1—O6	−111.18 (11)
C4—Fe1—C1—C9	51.38 (10)	C9—C8—K1—O5	80.62 (10)
C9—C1—C2—C3	−25.0 (2)	C7—C8—K1—O5	−42.94 (12)
Fe1—C1—C2—C3	54.32 (13)	C9—C8—K1—O2	−101.61 (10)
C9—C1—C2—Fe1	−79.35 (14)	C7—C8—K1—O2	134.83 (11)
C12—Fe1—C2—C3	−157.02 (10)	C9—C8—K1—O3	−162.66 (10)
C11—Fe1—C2—C3	−89.1 (2)	C7—C8—K1—O3	73.78 (11)
C16—Fe1—C2—C3	68.48 (11)	C9—C8—K1—O4	144.42 (9)
C15—Fe1—C2—C3	109.48 (10)	C7—C8—K1—O4	20.86 (11)
C4—Fe1—C2—C3	−31.50 (10)	C9—C8—K1—C7	123.56 (16)
C1—Fe1—C2—C3	−130.10 (14)	C9—C8—K1—C6	91.65 (11)
C12—Fe1—C2—C1	−26.92 (14)	C7—C8—K1—C6	−31.91 (10)
C11—Fe1—C2—C1	41.0 (3)	C9—C8—K1—C5	58.67 (10)
C16—Fe1—C2—C1	−161.42 (9)	C7—C8—K1—C5	−64.89 (11)
C3—Fe1—C2—C1	130.10 (14)	C6—C5—K1—O1	145.42 (11)
C15—Fe1—C2—C1	−120.42 (10)	C10—C5—K1—O1	21.06 (10)
C4—Fe1—C2—C1	98.60 (10)	C6—C5—K1—O6	−155.54 (11)
C1—C2—C3—C4	1.5 (2)	C10—C5—K1—O6	80.10 (10)
Fe1—C2—C3—C4	57.12 (13)	C6—C5—K1—O5	−93.22 (11)
C1—C2—C3—Fe1	−55.66 (13)	C10—C5—K1—O5	142.42 (11)
C12—Fe1—C3—C2	79.2 (3)	C6—C5—K1—O2	89.50 (11)
C11—Fe1—C3—C2	153.62 (10)	C10—C5—K1—O2	−34.86 (11)
C16—Fe1—C3—C2	−119.64 (10)	C6—C5—K1—O3	18.96 (11)
C15—Fe1—C3—C2	−82.59 (11)	C10—C5—K1—O3	−105.40 (10)
C4—Fe1—C3—C2	129.54 (15)	C6—C5—K1—O4	−41.51 (11)
C1—Fe1—C3—C2	30.94 (10)	C10—C5—K1—O4	−165.87 (10)
C12—Fe1—C3—C4	−50.3 (3)	C6—C5—K1—C7	32.05 (10)
C11—Fe1—C3—C4	24.08 (15)	C10—C5—K1—C7	−92.31 (11)
C16—Fe1—C3—C4	110.82 (10)	C10—C5—K1—C6	−124.36 (16)
C15—Fe1—C3—C4	147.87 (10)	C6—C5—K1—C8	65.84 (11)
C2—Fe1—C3—C4	−129.54 (15)	C10—C5—K1—C8	−58.52 (10)
C1—Fe1—C3—C4	−98.60 (11)	O6—K1—O1—C30	12.09 (9)
C2—C3—C4—C10	23.5 (2)	O5—K1—O1—C30	17.53 (11)
Fe1—C3—C4—C10	79.89 (13)	O2—K1—O1—C30	−145.04 (11)
C2—C3—C4—Fe1	−56.34 (13)	O3—K1—O1—C30	−147.96 (10)
C12—Fe1—C4—C3	163.98 (10)	O4—K1—O1—C30	−67.09 (16)
C11—Fe1—C4—C3	−163.72 (10)	C7—K1—O1—C30	128.75 (10)
C16—Fe1—C4—C3	−76.00 (11)	C6—K1—O1—C30	102.26 (11)
C15—Fe1—C4—C3	−53.04 (15)	C8—K1—O1—C30	135.07 (10)
C2—Fe1—C4—C3	31.22 (10)	C5—K1—O1—C30	87.59 (10)
C1—Fe1—C4—C3	70.55 (11)	O6—K1—O1—C19	154.46 (12)
C12—Fe1—C4—C10	42.54 (13)	O5—K1—O1—C19	159.91 (10)
C11—Fe1—C4—C10	74.83 (10)	O2—K1—O1—C19	−2.66 (10)
C16—Fe1—C4—C10	162.55 (10)	O3—K1—O1—C19	−5.58 (12)
C3—Fe1—C4—C10	−121.45 (15)	O4—K1—O1—C19	75.29 (17)
C15—Fe1—C4—C10	−174.49 (10)	C7—K1—O1—C19	−88.88 (12)
C2—Fe1—C4—C10	−90.23 (11)	C6—K1—O1—C19	−115.36 (11)
C1—Fe1—C4—C10	−50.90 (10)	C8—K1—O1—C19	−82.56 (11)
C10—C5—C6—C7	1.7 (3)	C5—K1—O1—C19	−130.04 (11)
K1—C5—C6—C7	−72.46 (15)	O1—K1—O2—C21	−153.62 (11)
C10—C5—C6—K1	74.20 (15)	O6—K1—O2—C21	−175.82 (9)
C5—C6—C7—C8	0.9 (3)	O5—K1—O2—C21	109.87 (15)
K1—C6—C7—C8	−78.03 (16)	O3—K1—O2—C21	23.44 (9)
C5—C6—C7—K1	78.91 (15)	O4—K1—O2—C21	44.00 (11)
C6—C7—C8—C9	−2.5 (3)	C7—K1—O2—C21	−45.06 (10)
K1—C7—C8—C9	−76.77 (15)	C6—K1—O2—C21	−51.82 (11)
C6—C7—C8—K1	74.31 (16)	C8—K1—O2—C21	−62.31 (10)
C7—C8—C9—C10	1.4 (2)	C5—K1—O2—C21	−80.10 (11)
K1—C8—C9—C10	−64.79 (13)	O1—K1—O2—C20	−30.31 (9)
C7—C8—C9—C1	−173.11 (16)	O6—K1—O2—C20	−52.52 (10)
K1—C8—C9—C1	120.69 (14)	O5—K1—O2—C20	−126.82 (14)
C2—C1—C9—C8	−162.24 (15)	O3—K1—O2—C20	146.75 (10)
Fe1—C1—C9—C8	131.52 (14)	O4—K1—O2—C20	167.31 (9)
C2—C1—C9—C10	23.1 (2)	C7—K1—O2—C20	78.25 (10)
Fe1—C1—C9—C10	−43.11 (14)	C6—K1—O2—C20	71.48 (10)
C6—C5—C10—C9	−2.7 (2)	C8—K1—O2—C20	60.99 (9)
K1—C5—C10—C9	60.69 (13)	C5—K1—O2—C20	43.21 (10)
C6—C5—C10—C4	171.20 (15)	O1—K1—O3—C23	147.96 (10)
K1—C5—C10—C4	−125.37 (14)	O6—K1—O3—C23	62.70 (16)
C8—C9—C10—C5	1.2 (2)	O5—K1—O3—C23	−17.99 (12)
C1—C9—C10—C5	176.05 (14)	O2—K1—O3—C23	145.06 (12)
C8—C9—C10—C4	−173.22 (14)	O4—K1—O3—C23	−13.44 (10)
C1—C9—C10—C4	1.7 (2)	C7—K1—O3—C23	−108.02 (11)
C3—C4—C10—C5	161.21 (15)	C6—K1—O3—C23	−91.28 (11)
Fe1—C4—C10—C5	−133.04 (14)	C8—K1—O3—C23	−132.41 (11)
C3—C4—C10—C9	−24.8 (2)	C5—K1—O3—C23	−98.89 (11)
Fe1—C4—C10—C9	41.00 (14)	O1—K1—O3—C22	15.21 (11)
C16—Fe1—C11—C12	114.26 (10)	O6—K1—O3—C22	−70.04 (16)
C3—Fe1—C11—C12	−155.64 (10)	O5—K1—O3—C22	−150.74 (10)
C15—Fe1—C11—C12	75.15 (10)	O2—K1—O3—C22	12.31 (10)
C2—Fe1—C11—C12	−86.6 (2)	O4—K1—O3—C22	−146.18 (11)
C4—Fe1—C11—C12	−140.34 (10)	C7—K1—O3—C22	119.24 (11)
C1—Fe1—C11—C12	−55.59 (12)	C6—K1—O3—C22	135.97 (11)
C12—Fe1—C11—C18	−117.39 (16)	C8—K1—O3—C22	94.84 (11)
C16—Fe1—C11—C18	−3.13 (12)	C5—K1—O3—C22	128.36 (10)
C3—Fe1—C11—C18	86.97 (14)	O1—K1—O4—C25	120.25 (14)
C15—Fe1—C11—C18	−42.24 (12)	O6—K1—O4—C25	49.17 (11)
C2—Fe1—C11—C18	156.0 (2)	O5—K1—O4—C25	25.78 (10)
C4—Fe1—C11—C18	102.27 (12)	O2—K1—O4—C25	−170.22 (10)
C1—Fe1—C11—C18	−172.98 (11)	O3—K1—O4—C25	−149.51 (11)
C18—C11—C12—C13	−1.4 (2)	C7—K1—O4—C25	−74.70 (11)
Fe1—C11—C12—C13	−106.05 (15)	C6—K1—O4—C25	−49.95 (11)
C18—C11—C12—Fe1	104.69 (15)	C8—K1—O4—C25	−83.23 (11)
C16—Fe1—C12—C11	−68.04 (10)	C5—K1—O4—C25	−34.13 (11)
C3—Fe1—C12—C11	92.7 (3)	O1—K1—O4—C24	−112.43 (15)
C15—Fe1—C12—C11	−104.24 (10)	O6—K1—O4—C24	176.48 (10)
C2—Fe1—C12—C11	154.72 (10)	O5—K1—O4—C24	153.09 (12)
C4—Fe1—C12—C11	53.76 (12)	O2—K1—O4—C24	−42.90 (12)
C1—Fe1—C12—C11	137.91 (10)	O3—K1—O4—C24	−22.19 (10)
C11—Fe1—C12—C13	116.98 (16)	C7—K1—O4—C24	52.62 (11)
C16—Fe1—C12—C13	48.94 (13)	C6—K1—O4—C24	77.37 (11)
C3—Fe1—C12—C13	−150.3 (2)	C8—K1—O4—C24	44.09 (12)
C15—Fe1—C12—C13	12.74 (12)	C5—K1—O4—C24	93.18 (11)
C2—Fe1—C12—C13	−88.29 (14)	O1—K1—O5—C26	−152.12 (11)
C4—Fe1—C12—C13	170.74 (11)	O6—K1—O5—C26	−146.68 (12)
C1—Fe1—C12—C13	−105.10 (12)	O2—K1—O5—C26	−64.62 (18)
C11—C12—C13—C14	57.8 (2)	O3—K1—O5—C26	13.57 (12)
Fe1—C12—C13—C14	−22.33 (18)	O4—K1—O5—C26	8.98 (11)
C12—C13—C14—C15	21.5 (2)	C7—K1—O5—C26	90.30 (12)
C13—C14—C15—C16	−90.26 (19)	C6—K1—O5—C26	99.05 (12)
C13—C14—C15—Fe1	−11.36 (18)	C8—K1—O5—C26	106.87 (11)
C12—Fe1—C15—C16	116.31 (11)	C5—K1—O5—C26	124.11 (12)
C11—Fe1—C15—C16	76.95 (10)	O1—K1—O5—C33	−15.23 (11)
C3—Fe1—C15—C16	−68.72 (11)	O6—K1—O5—C33	−9.79 (9)
C2—Fe1—C15—C16	−108.57 (11)	O2—K1—O5—C33	72.27 (17)
C4—Fe1—C15—C16	−35.54 (15)	O3—K1—O5—C33	150.46 (9)
C1—Fe1—C15—C16	−146.77 (10)	O4—K1—O5—C33	145.87 (11)
C12—Fe1—C15—C14	−0.58 (12)	C7—K1—O5—C33	−132.82 (10)
C11—Fe1—C15—C14	−39.94 (13)	C6—K1—O5—C33	−124.07 (10)
C16—Fe1—C15—C14	−116.89 (16)	C8—K1—O5—C33	−116.25 (10)
C3—Fe1—C15—C14	174.39 (12)	C5—K1—O5—C33	−99.00 (10)
C2—Fe1—C15—C14	134.54 (12)	O1—K1—O6—C31	22.09 (9)
C4—Fe1—C15—C14	−152.43 (12)	O5—K1—O6—C31	−152.57 (10)
C1—Fe1—C15—C14	96.34 (13)	O2—K1—O6—C31	43.99 (10)
C14—C15—C16—C17	3.0 (2)	O3—K1—O6—C31	117.20 (13)
Fe1—C15—C16—C17	−102.06 (16)	O4—K1—O6—C31	−175.47 (9)
C14—C15—C16—Fe1	105.03 (15)	C7—K1—O6—C31	−73.40 (10)
C12—Fe1—C16—C15	−65.50 (11)	C6—K1—O6—C31	−89.36 (10)
C11—Fe1—C16—C15	−102.89 (10)	C8—K1—O6—C31	−46.19 (10)
C3—Fe1—C16—C15	119.79 (10)	C5—K1—O6—C31	−79.46 (10)
C2—Fe1—C16—C15	83.71 (11)	O1—K1—O6—C32	149.82 (10)
C4—Fe1—C16—C15	158.04 (10)	O5—K1—O6—C32	−24.84 (9)
C1—Fe1—C16—C15	62.09 (16)	O2—K1—O6—C32	171.72 (9)
C12—Fe1—C16—C17	55.10 (13)	O3—K1—O6—C32	−115.07 (13)
C11—Fe1—C16—C17	17.71 (12)	O4—K1—O6—C32	−47.74 (10)
C3—Fe1—C16—C17	−119.61 (13)	C7—K1—O6—C32	54.33 (10)
C15—Fe1—C16—C17	120.60 (17)	C6—K1—O6—C32	38.37 (10)
C2—Fe1—C16—C17	−155.69 (12)	C8—K1—O6—C32	81.55 (10)
C4—Fe1—C16—C17	−81.36 (13)	C5—K1—O6—C32	48.27 (9)
C1—Fe1—C16—C17	−177.31 (12)	C30—O1—C19—C20	177.53 (13)
C15—C16—C17—C18	51.3 (2)	K1—O1—C19—C20	33.64 (17)
Fe1—C16—C17—C18	−29.01 (19)	C21—O2—C20—C19	−176.10 (13)
C12—C11—C18—C17	−91.52 (19)	K1—O2—C20—C19	61.35 (13)
Fe1—C11—C18—C17	−12.05 (18)	O1—C19—C20—O2	−63.86 (17)
C16—C17—C18—C11	26.9 (2)	C20—O2—C21—C22	−178.76 (13)
C6—C7—K1—O1	−106.36 (10)	K1—O2—C21—C22	−57.35 (14)
C8—C7—K1—O1	15.32 (12)	C23—O3—C22—C21	−179.16 (13)
C6—C7—K1—O6	−39.48 (12)	K1—O3—C22—C21	−45.48 (15)
C8—C7—K1—O6	82.20 (11)	O2—C21—C22—O3	70.65 (16)
C6—C7—K1—O5	20.15 (11)	C22—O3—C23—C24	−179.81 (14)
C8—C7—K1—O5	141.83 (10)	K1—O3—C23—C24	46.89 (16)
C6—C7—K1—O2	−166.17 (10)	C25—O4—C24—C23	−178.44 (13)
C8—C7—K1—O2	−44.50 (11)	K1—O4—C24—C23	54.97 (15)
C6—C7—K1—O3	137.27 (11)	O3—C23—C24—O4	−69.17 (17)
C8—C7—K1—O3	−101.06 (11)	C24—O4—C25—C26	175.34 (13)
C6—C7—K1—O4	78.00 (10)	K1—O4—C25—C26	−57.72 (14)
C8—C7—K1—O4	−160.32 (11)	C33—O5—C26—C25	−178.84 (13)
C8—C7—K1—C6	121.68 (16)	K1—O5—C26—C25	−40.89 (16)
C6—C7—K1—C8	−121.68 (16)	O4—C25—C26—O5	65.82 (17)
C6—C7—K1—C5	−30.64 (9)	C19—O1—C30—C31	171.64 (13)
C8—C7—K1—C5	91.03 (12)	K1—O1—C30—C31	−43.07 (15)
C7—C6—K1—O1	83.65 (11)	C32—O6—C31—C30	179.12 (13)
C5—C6—K1—O1	−38.36 (12)	K1—O6—C31—C30	−53.02 (14)
C7—C6—K1—O6	146.61 (10)	O1—C30—C31—O6	63.81 (16)
C5—C6—K1—O6	24.60 (11)	C31—O6—C32—C33	−174.09 (13)
C7—C6—K1—O5	−159.10 (11)	K1—O6—C32—C33	57.49 (13)
C5—C6—K1—O5	78.89 (10)	C26—O5—C33—C32	−179.00 (13)
C7—C6—K1—O2	16.05 (12)	K1—O5—C33—C32	41.47 (14)
C5—C6—K1—O2	−105.96 (11)	O6—C32—C33—O5	−66.62 (16)
